# Machine learning classifiers and associations of cognitive performance with hippocampal subfields in amnestic mild cognitive impairment

**DOI:** 10.3389/fnagi.2023.1273658

**Published:** 2023-11-30

**Authors:** Qi Feng, Luoyu Wang, Xue Tang, Xiuhong Ge, Hanjun Hu, Zhengluan Liao, Zhongxiang Ding

**Affiliations:** ^1^Department of Radiology, Hangzhou First People’s Hospital, Hangzhou, China; ^2^School of Medical Imaging, Hangzhou Medical College, Hangzhou, China; ^3^Zhejiang Chinese Medical University, Hangzhou, China; ^4^Department of Psychiatry, Zhejiang Provincial People’s Hospital/People’s Hospital of Hangzhou Medical College, Hangzhou, China

**Keywords:** Alzheimer’s disease, amnestic mild cognitive impairment, magnetic resonance imaging, hippocampal subfields, machine learning

## Abstract

**Background:**

Neuroimaging studies have demonstrated alterations in hippocampal volume and hippocampal subfields among individuals with amnestic mild cognitive impairment (aMCI). However, research on using hippocampal subfield volume modeling to differentiate aMCI from normal controls (NCs) is limited, and the relationship between hippocampal volume and overall cognitive scores remains unclear.

**Methods:**

We enrolled 50 subjects with aMCI and 44 NCs for this study. Initially, a univariate general linear model was employed to analyze differences in the volumes of hippocampal subfields. Subsequently, two sets of dimensionality reduction methods and four machine learning techniques were applied to distinguish aMCI from NCs based on hippocampal subfield volumes. Finally, we assessed the correlation between the relative volumes of hippocampal subfields and cognitive test variables (Mini-Mental State Examination (MMSE) and Montreal Cognitive Assessment Scale (MoCA)).

**Results:**

Significant volume differences were observed in several hippocampal subfields, notably in the left hippocampus. Specifically, the volumes of the hippocampal tail, subiculum, CA1, presubiculum, molecular layer, GC-ML-DG, CA3, CA4, and fimbria differed significantly between the two groups. The highest area under the curve (AUC) values for left and right hippocampal machine learning classifiers were 0.678 and 0.701, respectively. Moreover, the volumes of the left subiculum, left molecular layer, right subiculum, right CA1, right molecular layer, right GC-ML-DG, and right CA4 exhibited the strongest and most consistent correlations with MoCA scores.

**Conclusion:**

Hippocampal subfield volume may serve as a predictive marker for aMCI. These findings underscore the sensitivity of hippocampal subfield volume to overall cognitive performance.

## Introduction

Alzheimer’s disease (AD) is characterized by a prolonged prodromal phase, spanning from the preclinical stage to the prodromal stage before progressing to full-blown AD (Liu et al., 2021). Amnestic mild cognitive impairment (aMCI) represents a level of memory loss and cognitive decline in elderly individuals that falls short of dementia criteria and is considered a risk factor for Alzheimer’s disease (Oltra-Cucarella et al., 2018). Currently, clinical and research settings employ positron emission computed tomography (PET) imaging and the measurement of amyloid-β 42 (Aβ42) and Tau protein concentrations in cerebrospinal fluid as diagnostic biomarkers for AD. However, the high cost and radiation associated with PET scans limit their clinical utility. Cerebrospinal fluid examination via lumbar puncture, while providing valuable insights, carries the risks of local anesthetic allergies and intracranial infections. In contrast, structural magnetic resonance imaging (MRI) offers a non-invasive and highly repeatable alternative.

The hippocampus is an important brain region in the central nervous system involved in learning and memory storage. Hippocampal atrophy is one of the most effective, convenient, and widely used biomarkers for the clinical diagnosis of AD (Flores et al., 2015). Recent advancements in neuroimaging have revealed abnormal intracranial structures in AD patients, notably the reduction in hippocampal volume. Our previous research has identified disparities in the imaging characteristics of the left and right hippocampi in aMCI patients compared to normal controls, suggesting that hippocampal imaging features hold promise as potential biomarkers for aMCI and AD diagnosis (Feng et al., 2019). The hippocampus is an important part of the memory system, which is composed of different subfields with different functional characteristics, and each subregion is affected differently in the course of AD (Tamnes et al., 2014). West et al. (1994) reported a substantial neuronal loss, reaching up to 68%, in the hippocampal CA1 region of AD patients. Accurate measurement of hippocampal subfield volumes is of paramount importance for early diagnosis, and further advancements in this domain, including optimization, standardization, and automation of techniques, are imperative (Chételat, 2018).

Numerous methods have been employed to investigate hippocampal subfields in the context of AD spectrum diseases, including stereoscopic brain imaging, radial atrophy assessment, and the voxel-based morphometry (VBM) technique. One study examining hippocampal subfields in AD revealed that as the disease advances, the volume of both the hippocampus and its subfields (e.g., CA1, subiculum, presubiculum, molecular layer, and fimbria) gradually diminishes, with the left hippocampus exhibiting particularly pronounced changes (Zhao et al., 2019). Hari et al. (2022) found that compared with the MCI group and the subjective cognitive impairment (SCI) group, the volume of bilateral CA1, dentate gyrus DG, and subiculum decreased in the AD group; no significant volume change was found between the SCI and MCI groups. Boccardi et al. (2018) identified significant volume differences in 11 left hippocampal subfields (excluding the parasubiculum) among the AD, MCI, and NC groups, as well as in all 12 right hippocampal subfields among these groups. Moreover, Huang et al. (2022) found atrophy in CA1, molecular layer, subiculum, GC-ML-DG, CA4, and CA3 in the aMCI group, but the left and right hemispheres were not studied separately. However, studies have indicated that the asymmetry of hippocampal subfield atrophy in AD and MCI exists (Alessia et al., 2018; Jahanshahi et al., 2023). There is no consensus on how the hippocampal subfield atrophies.

There are relatively few studies based on hippocampal subfield volume modeling to distinguish aMCI from NC. Guo et al. (2020) applied FreeSurfer software version 5.0.0 to divide the bilateral hippocampus into 16 subregions. The achieved accuracy rates were 64.62%, 78.96%, and 70.33% for the support vector machine (SVM) classifier based on hippocampal subfield volumes in distinguishing stable MCI-NC, converted MCI-NC, and converted MCI-stable MCI cases, respectively. However, their study employed only a single machine learning method. Qu et al. (2023) found the volumes of the right subiculum, CA1, molecular layer, whole hippocampus, whole amygdala, basal, and accessory basal were significantly larger in NC compared to the MCI group. They achieved an area under the curve (AUC) of 0.79 in the integrated receiver operating characteristic (ROC) analysis for discriminating between MCI and NC; however, cross-validation was not conducted in their study.

Several studies have made attempts to uncover the intrinsic connection between the hippocampus or its regions and memory, suggesting their potential involvement in early changes or progression of AD. In one correlational analysis, it was found that within the aMCI group, volumes of the subiculum, presubiculum, and CA4/dentate gyrus were associated with delayed recall scores, and volumes of the subiculum and CA4/dentate gyrus correlated with informant-reported memory difficulties (O’Shea et al., 2022). Huang et al. (2022) found that in the aMCI group, visual memory scores were positively correlated with CA1, molecular layer, subiculum, and GC-ML-DG volumes. Zhao et al. (2019) found that the left subiculum showed a positive correlation with memory scores. However, employing a combination of multiple memory tests can be burdensome for elderly individuals and may not be suitable for routine clinical studies. Meanwhile, the Montreal Cognitive Assessment Scale (MoCA) offers a quick means of cognitive assessment that encompasses executive function, visuospatial recognition, memory, and other cognitive domains.

To address these considerations, this study pursued three primary objectives: (1) investigate changes in hippocampal subfield volumes in aMCI patients utilizing 3D T1 structural MRI and FreeSurfer 6.03 software’s detailed partitioning method; (2) develop machine learning classification models grounded in hippocampal subfield volumes; and (3) analyze the correlation between hippocampal subfield volumes and cognitive function scores, with a specific focus on MoCA.

## Materials and methods

### Patient population and data acquisition

Between September 2016 and August 2020, we recruited a cohort comprising 50 individuals with aMCI and 44 normal control (NC) subjects. These participants were sourced from the Health Promotion Center and the Psychiatry Department of Zhejiang Provincial People’s Hospital. The study received approval from the Ethics Committee of Zhejiang Provincial People’s Hospital (2012KY002) and adhered to the principles outlined in the Declaration of Helsinki. Informed consent forms were duly signed either by the subjects or their legal representatives.

All subjects underwent laboratory tests, physical examinations, and a 3.0 T MR scan. The inclusion criteria for aMCI subjects were as follows: self-reported memory impairment as the primary complaint; clinically normal manifestations; Mini-Mental State Examination (MMSE) score >24 but ≤27 (Mckhann et al., 2011). Inclusion criteria for NC subjects: no neurological impairment such as hearing or vision deficits; no history of stroke, epilepsy, depression, or other neurological or psychiatric disorders; conventional brain MR imaging revealing no evidence of infarction, hemorrhage, tumors, or other abnormalities; MMSE score ≥28. Exclusion criteria included severe anemia, hypertension, diabetes, or other systemic diseases; a history of brain trauma; neurological conditions such as stroke, brain tumors, Parkinson’s disease, epilepsy, or other disorders known to cause memory impairments; a history of mental illness or the use of psychotropic medications; contraindications for MRI scanning; and medial temporal lobe signal abnormalities detected on MRI fluid attenuated inversion recovery (FLAIR) or T2-weighted imaging (T2WI) images attributed to infectious or vascular factors.

Based on these inclusion and exclusion criteria, we initially enrolled 60 aMCI patients and 46 NCs but subsequently excluded a few cases due to unsuccessful MRI image acquisition (4 aMCI patients and 1 NC subject). Ultimately, our study included 50 aMCI patients and 44 NC subjects.

### Neuropsychological scale tests

The comprehensive assessment of cognitive function encompassed two components: (1) MMSE: this evaluation covered various domains, including orientation, memory, attention, numeracy, language abilities, and visuospatial cognition, with a maximum score of 30. (2) MoCA-Basic Edition (MoCA-B): This assessment addressed attention and concentration, executive function, memory, language skills, visual-spatial abilities, abstract thinking, computation, and orientation. The assessments were conducted in a quiet environment, assuming the patients had clear consciousness and were free from inhibitory psychology. The maximum score attainable on this scale was 30, with higher scores indicating better cognitive function.

### Image acquisition

All imaging procedures were conducted using a 3.0 T MR scanner (Discovery MR750; GE Healthcare, Waukesha, WI, United States). Routine MRI scans were performed on all subjects to exclude other conditions that might cause structural brain changes. High-resolution 3D T1-weighted magnetization-prepared rapid gradient echo (T1-MPRAGE) sequences were acquired with the following parameters: repetition time (TR) = 6.7 ms, echo time (TE) = 2.9 ms, inversion time (TI) = 450 ms, flip angle = 12°, field of view (FOV) = 256 × 256 mm^2^, slice thickness/slice gap = 1/0 mm, matrix = 256 × 256, with a total of 192 sagittal sections. Subjects’ heads were securely positioned, and earplugs were inserted to minimize head movement.

### Hippocampal subfields segmentation

**Figure 1:**
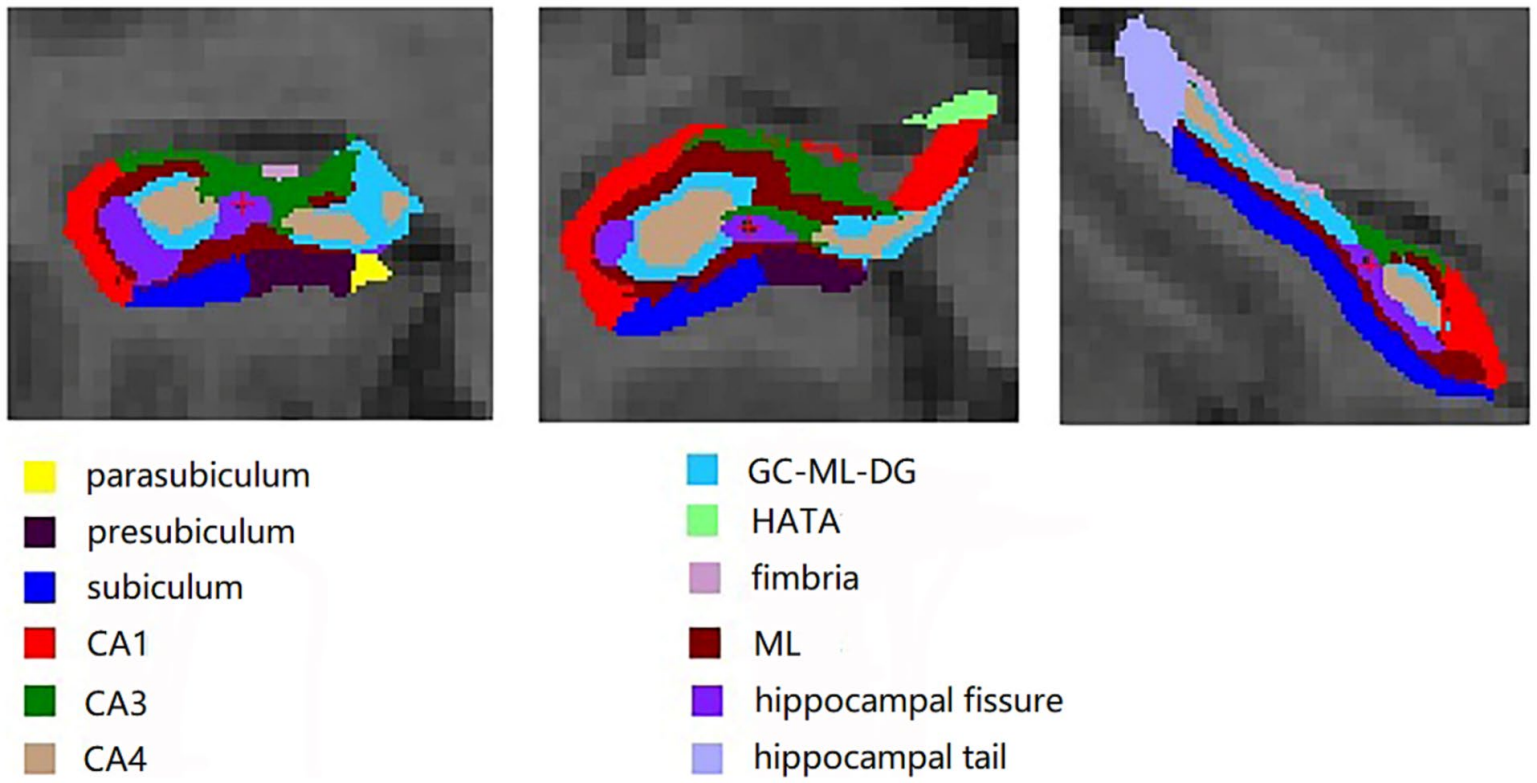
Twelve hippocampal subfields segmented on FreeSurfer software package: parasubiculum, presubiculum, subiculum, CA1, CA3, CA4, the molecular and granule cell layers of the dentate gyrus (GC-ML-DG), hippocampus-amygdala transition area (HATA), fimbria, molecular layer (ML), hippocampal fissure, and hippocampal tail.

The FreeSurfer software package (version 6.0.0, http://freesurfer.net/) was utilized to process the 3D T1-MPRAGE images. These methods have been described previously (Fischl et al., 2002, 2004; Segonne et al., 2004). The processing steps were as follows: (1) non-brain tissue was removed through cephalometric correction. (2) Registration in the Talairach standard space was conducted. (3) Cortical and subcortical structures were segmented using probabilistic brain maps. (4) Estimation of the estimated total intracranial volume (eTIV) for each subject was carried out through a standard FreeSurfer processing pipeline, employing the relationship between intracranial volume and the linear transformation of the map template (Buckner et al., 2004). (5) Bilateral hippocampal subfields were segmented using FreeSurfer’s built-in module, with a Bayesian statistical model incorporating a Markov random field prior used to estimate the label of each subfield. (6) An enclosure containing a seahorse was upsampled to an isotropic resolution of 0.5 mm. (7) Automatic division of the left and right hippocampus into 12 subfields, including parasubiculum, presubiculum, subiculum, CA1, CA3, CA4, the molecular and granule cell layers of the dentate gyrus (GC-ML-DG), hippocampus-amygdala transition area (HATA), fimbria, molecular layer (ML), hippocampal fissure, and hippocampal tail (Figure 1), was accomplished by maximizing the posterior probability of splitting. Finally, the total volume of the hippocampus and the volume of each subfield were extracted for further analysis.

### Statistical analysis

Statistical analysis was conducted using SPSS (version 22.0). All statistical tests were two-tailed. The data for demographic variables were subjected to a chi-square test. Continuous demographic variables were analyzed through a two-sample T-test. In this study, eTIV was used as a covariate to control for head size. Both the left and right hippocampi were analyzed separately. Univariate general linear models were employed to assess volume differences in hippocampal subfields, with age, sex, years of education, and eTIV included as covariates. These analyses were further adjusted using the Bonferroni correction. For correlation analysis, the relative volume of hippocampal subfields was initially calculated, representing the ratio of the volume of each subregion to eTIV. Subsequently, correlation assessments were performed between the relative volume of hippocampal subfields and cognitive test variables (MMSE and MoCA). These analyses were also corrected for multiple comparisons using Bonferroni correction, with a significance level of 0.05.

### Machine learning models’ construction

Two sets of four dimensionality reduction methods, namely fisher score (FSCR), RELF-F, maximum relevance and minimum redundancy (MRMR), and conditional mutual information metric (CMIM), were combined with four machine learning methods: linear discriminant analysis (LDA), logistic regression (LR), naive Bayes (NB), and SVM. These combinations were employed to distinguish between aMCI and NC based on the relative volumes of the 12 hippocampal subfields. Subsequently, a 5-fold cross-validation procedure was applied. A total of 16 classifiers were constructed for each of the left and right hippocampi. These procedures were executed using MATLAB 2018b.

## Results

### Comparison of demographic and neuropsychological performance

There were no significant differences in age, sex, or education between the aMCI patients and NC subjects (*p* > 0.05). However, there were significant differences in cognitive scores (MMSE and MoCA) between the two groups (*p* < 0.01) (Table 1).

**Table 1:** Demographic characteristics of aMCI and normal control groups.

	aMCI group	NC group	Statistic	*p*-value
Sample size	50	44	NA	NA
Age (years, mean ± SD)	65.84 ± 11.17	65.48 ± 9.69	0.17	0.87
Gender (male: female)	27:23	20:24	0.68^*^	0.41^*^
Education (years, mean ± SD)	7.12 ± 4.06	7.11 ± 3.36	0.01	0.99
MMSE	26.20 ± 0.88	29.02 ± 0.90	−17.92	<0.01
MoCA	22.44 ± 2.36	27.45 ± 1.37	−12.78	<0.01

SD, standard deviation; unless otherwise indicated, statistics were calculated with *t*-tests; ^*^χ^2^ test was used; MMSE, Mini-Mental State Examination; MoCA, Montreal Cognitive Assessment Scale.

### Comparisons of hippocampal subfield volumes

**Figure 2:**
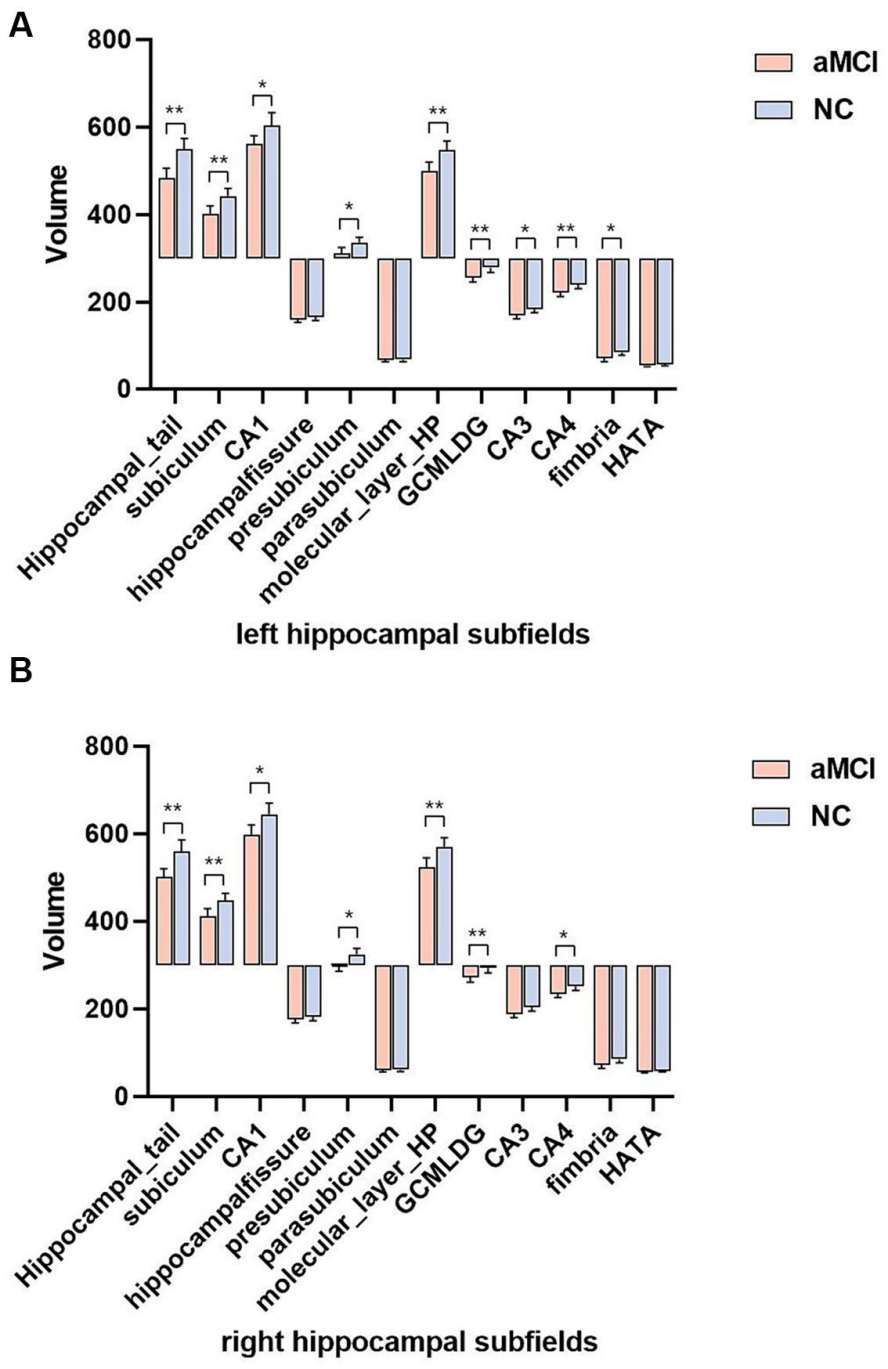
**(A,B)** Comparison of hippocampal subfields volume in aMCI and normal controls using Bonferroni corrected. ^*^p < 0.05 and ^**^p < 0.01. aMCI, amnestic mild cognitive impairment; NC, normal controls.

Table 2 displays the volumes and percentages of the 12 hippocampal subfields in both groups. In the left hippocampus, the volumes of the hippocampal tail, subiculum, CA1, presubiculum, molecular layer, GC-ML-DG, CA3, CA4, and fimbria exhibited significant differences between the two groups (*p* < 0.05, Bonferroni corrected) (Figure 2A). Similarly, in the right hippocampus, the volumes of the hippocampal tail, subiculum, CA1, presubiculum, molecular layer, GC-ML-DG, and CA4 showed significant differences between the two groups (*p* < 0.05, Bonferroni corrected) (Figure 2B).

**Table 2:** Comparison of hippocampal subfield volumes between patients with aMCI and normal controls.

	Left hemisphere		Right hemisphere	
	aMCI	NC	aMCI	NC
Hippocampal_tail	482.705 ± 80.722 (0.033%)^**^	549.229 ± 81.404 (0.038%)	501.597 ± 66.002 (0.034%)^**^	559.250 ± 87.532 (0.038%)
Subiculum	402.379 ± 61.609 (0.027%)^**^	442.309 ± 56.345 (0.030%)	412.293 ± 58.030 (0.028%)^**^	447.390 ± 54.578 (0.031%)
CA1	560.950 ± 68.110 (0.038%)^*^	603.848 ± 96.940 (0.041%)	598.164 ± 77.179 (0.040%)^*^	644.078 ± 84.479 (0.044%)
Hippocampal fissure	159.653 ± 27.231 (0.011%)	166.207 ± 26.576 (0.011%)	175.546 ± 27.179 (0.012%)	181.353 ± 27.440 (0.012%)
Presubiculum	311.758 ± 46.995 (0.021%)^*^	336.220 ± 39.341 (0.023%)	298.922 ± 27.423 (0.020%)^*^	324.587 ± 44.253 (0.022%)
Parasubiculum	65.844 ± 15.161 (0.004%)	66.965 ± 15.271 (0.005%)	59.579 ± 11.767 (0.004%)	61.008 ± 12.317 (0.004%)
Molecular_layer_HP	500.142 ± 67.718 (0.034%)^**^	546.876 ± 70.313 (0.037%)	524.365 ± 72.394 (0.035%)^**^	570.585 ± 66.949 (0.039%)
GC-ML-DG	256.648 ± 31.180 (0.017%)^**^	278.868 ± 37.804 (0.019%)	271.593 ± 36.230 (0.018%)^**^	293.844 ± 38.087 (0.020%)
CA3	169.411 ± 24.777 (0.011%)^*^	184.282 ± 26.477 (0.013%)	188.454 ± 28.717 (0.013%)	203.572 ± 27.298 (0.014%)
CA4	221.620 ± 29.946 (0.015%)^**^	240.300 ± 31.228 (0.016%)	234.615 ± 28.377 (0.016%)^*^	252.309 ± 31.754 (0.017%)
Fimbria	69.456 ± 24.788 (0.005%)^*^	83.327 ± 20.959 (0.006%)	71.544 ± 25.543 (0.005%)	84.723 ± 25.371 (0.006%)
HATA	52.983 ± 8.110 (0.004%)	55.583 ± 8.841 (0.004%)	55.588 ± 7.691 (0.004%)	58.625 ± 8.333 (0.004%)

The value is expressed in the form of a mean ± standard deviation for hippocampal subfield volumes in mm^3^. Values in parentheses = the mean of the subfield volume/eTIV. eTIV, estimated total intracranial volume; aMCI, amnestic mild cognitive impairment; NC, normal control. ^*^Compared with the NC group, *p* < 0.05; ^**^compared with NC group, *p* < 0.01.

### Machine learning results

**Figure 3:**
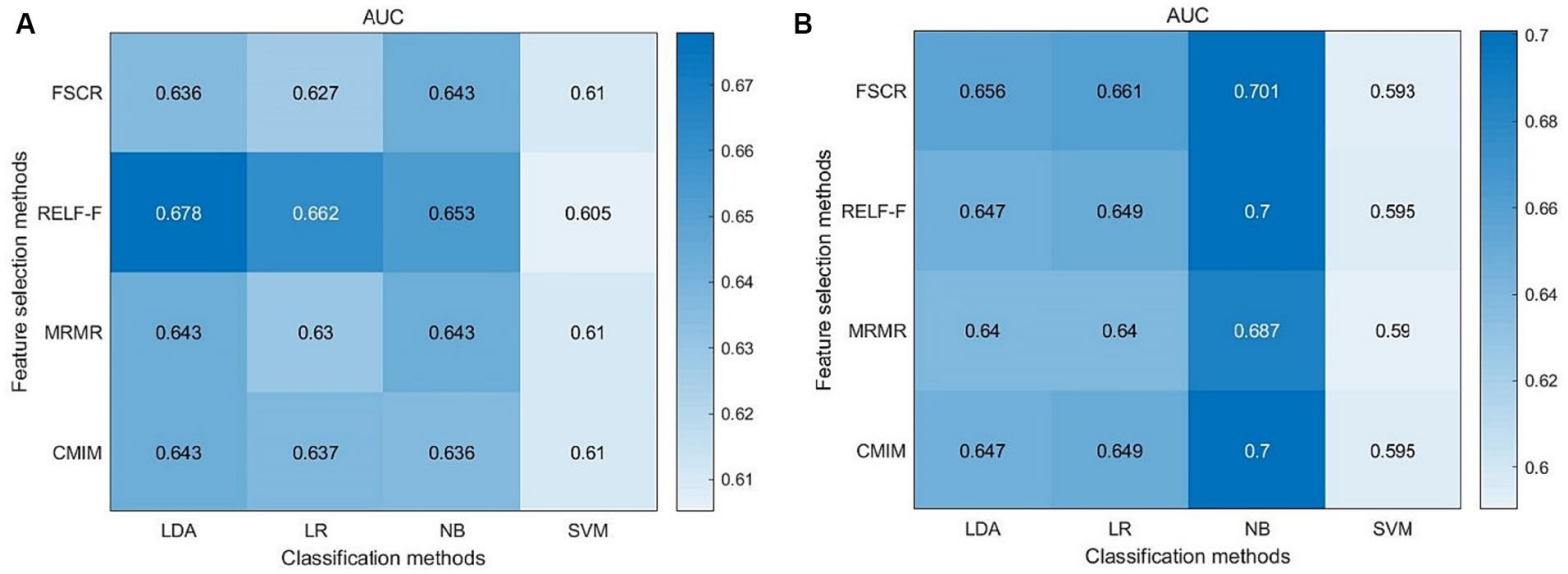
Machine learning results of the left hippocampus **(A)** and right hippocampus **(B)**.

The highest area under the curve (AUC) value in the left hippocampal model was 0.678, achieved through a combination of RELF-F and LDA (Figure 3A). In the right hippocampal model, the highest AUC value was 0.701, achieved through a combination of FSCR and NB (Figure 3B).

### Relationship between MMSE, MoCA, and hippocampal subfield volumes

**Figure 4:**
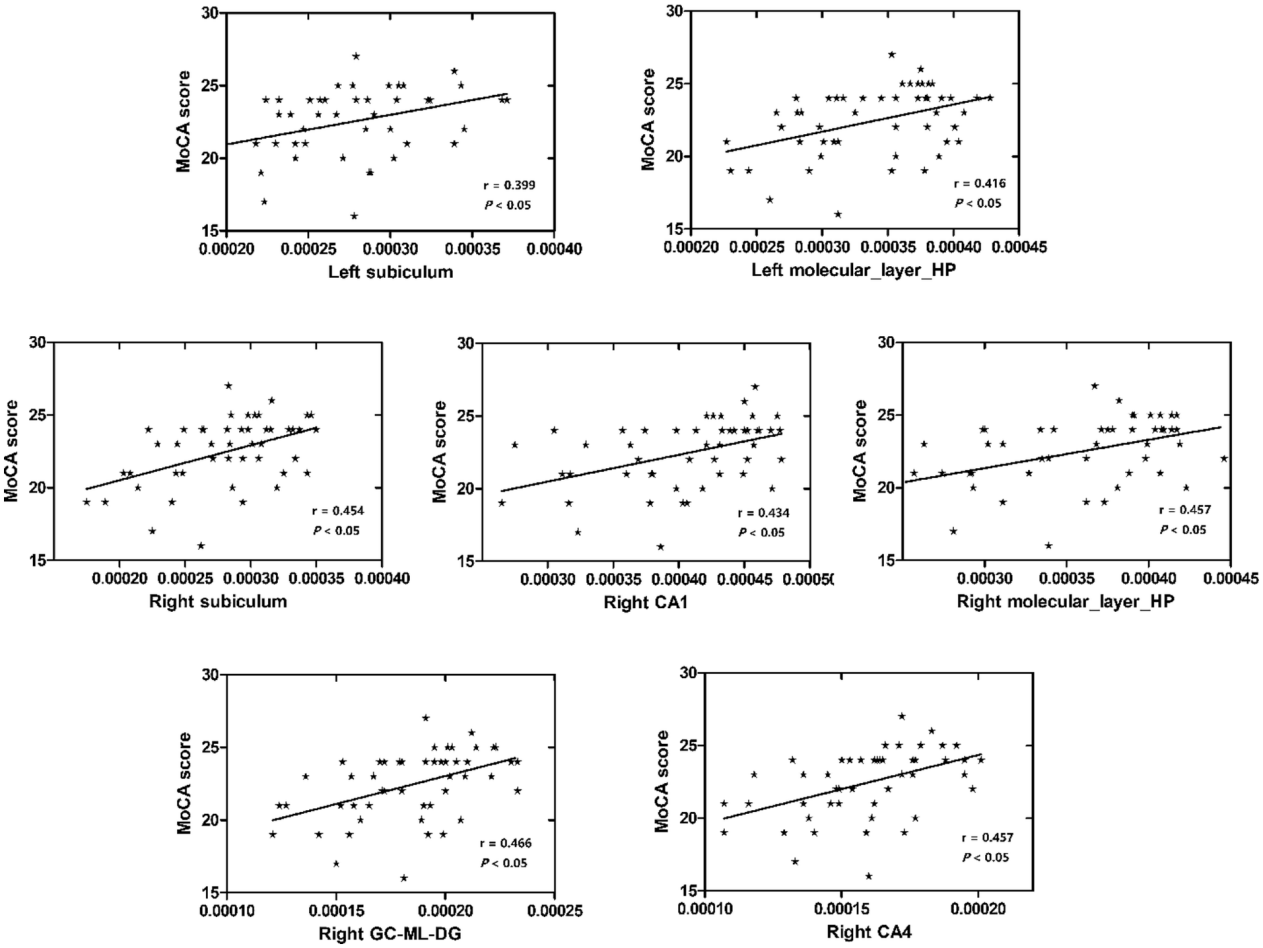
Relationship between MoCA scores and hippocampal subfield volumes.

No significant correlation was observed between MMSE scores and the volumes of bilateral hippocampal subfields (*p* > 0.05, Bonferroni correction). left subiculum, left molecular layer, right subiculum, right CA1, right molecular layer, right GC-ML-DG, and right CA4 were found to exhibit the strongest and most significant correlations with MoCA scores (*p* < 0.05, Bonferroni correction) (Table 3 and Figure 4).

**Table 3:** Correlation between relative volumes of hippocampal subregions and cognitive test scores (MMSE, MoCA).

	Left hemisphere		Right hemisphere	
	MMSE	MoCA	MMSE	MoCA
Hippocampal_tail	0.360	0.308	0.352	0.260
Subiculum	0.282	0.399^*^	0.345	0.454^*^
CA1	0.333	0.338	0.295	0.434^*^
Hippocampal fissure	0.006	0.208	0.125	0.256
Presubiculum	0.349	0.363	0.359	0.308
Parasubiculum	0.234	0.125	0.160	0.209
Molecular_layer_HP	0.390	0.416^*^	0.357	0.457^*^
GC-ML-DG	0.379	0.388	0.369	0.466^*^
CA3	0.324	0.370	0.216	0.395
CA4	0.387	0.390	0.356	0.457^*^
Fimbria	0.127	0.209	0.304	0.306
HATA	0.326	0.051	0.274	0.294

The values in the table are the correlation coefficients. ^*^(*p* < 0.05, Bonferroni corrected).

## Discussion

This study investigated the change in volume in aMCI patients and established machine learning classification models based on the hippocampal subfield volume. The volumes of hippocampal tail, subiculum, CA1, presubiculum, molecular layer, GC-ML-DG, CA3, CA4, and fimbria were significantly different between the two groups, especially in the left hippocampus. The highest area under curve (AUC) value in the left and right hippocampal machine learning classifiers was 0.678 and 0.701, respectively. The volume of the left subiculum, left molecular layer, right subiculum, right CA1, right molecular layer, right GC-ML-DG, and right CA4 was most strongly and actively correlated with MoCA scores.

Hippocampal atrophy is widely recognized as an early and specific marker in AD patients. Neuroimaging studies have consistently reported varying degrees of involvement of the hippocampus and its subfields in AD and early AD (Chételat, 2018). In the volume studies of hippocampal subregions in AD patients, the atrophy of the CA1 region is a relatively consistent result (Flores et al., 2015). There have been many studies on hippocampal subfield volume using FreeSurfer software. It is commonly found that the volume of these subfields of the CA1, subiculum, presubiculum, molecular layer, GC-ML-DG, CA4, CA3, and fimbria decreases in MCI (Boccardi et al., 2018; Zhao et al., 2019; Hari et al., 2022; Huang et al., 2022). In our study, significant differences in the volumes of the hippocampal tail, subiculum, CA1, presubiculum, molecular layer, GC-ML-DG, CA3, CA4, and fimbria between the aMCI and NC groups were observed, with a pronounced effect noted in the left hippocampus. In terms of neuron loss, one study reported that the main subfields of loss in AD patients were CA1 (68%) and subiculum (47%) compared to controls (25%) (West et al., 1994). The presubiculum plays a unique role in AD; these studies showed that “lake-like” amyloid beta (Aβ) deposits appear under the presubiculum early in the disease process (Murray et al., 2018). Interneurons and synaptic connections in the molecular layer are involved in the regulation of activity in the hippocampus. Volumetric studies showed that the volume reduction mainly occurred in CA1, but also involved CA3/DG (Yassa et al., 2010), CA4/DG (Pluta et al., 2012). Fimbria plays an important role in executive function (Seiger et al., 2021). These results are consistent with most of the aforementioned findings, and a novel subregion, the hippocampal tail, is introduced, which appears to play a distinctive role in aMCI patients (Peter et al., 2018). The hippocampal tail is often associated with verbal memory. Parker et al. reported significant volume declines in CA1 and hippocampal tail in AD patients (Parker et al., 2018). Additionally, previous studies have indicated the importance of the right hippocampal tail in SCD patients, with significant reductions in gray matter volume (Liang et al., 2020).

There are relatively few studies based on hippocampal subfield volume modeling to distinguish aMCI from NC. One study showed that the accuracy of an SVM classifier based on hippocampal subfield volumes was 64.62% for stable MCI-NC, 78.96% for converted MCI-NC, and 70.33% for converted MCI-stable MCI (Guo et al., 2020). However, it used only one machine learning method. Qu et al. (2023) found the AUC was 0.79 for the identification of MCI and NC, but no cross-validation was performed. In the present study, the highest AUC values achieved for the left and right hippocampal machine learning classifiers were 0.678 and 0.701, respectively. Although the classification accuracy of the model is not very high, it also provides meaningful information, which can only say that the trend of hippocampal subfield volume change has diagnostic significance for aMCI, but cannot be said to have clinical value. The clinical value needs to be further studied.

Although there have been many studies showing that hippocampal subfield volume is associated with various memory scores (Zammit et al., 2017; Zhao et al., 2019; Huang et al., 2022; O’Shea et al., 2022). MoCA is a cognitive assessment that can measure executive function, visuospatial recognition, memory, and other cognitive functions, which is very important for the cognitive assessment of AD and aMCI patients. In our investigation, we identified that the volumes of the left subiculum, left molecular layer, right subiculum, right CA1, right molecular layer, right GC-ML-DG, and right CA4 were the most strongly and significantly correlated with MoCA scores. Liang et al. (2018) found that in MCI patients, left subiculum and presubiculum volumes were positively correlated with MoCA scores, but statistical *p*-values were not corrected. A study of AD and MCI in Japan Ogawa et al. (2019) found that the Japanese version of the MoCA had a higher correlation with the subfield volume of CA1, DG, subiculum, and entorhinal cortex than the MMSE. However, the sample size was relatively small. One study has found that some hippocampal subfield volumes were significantly correlated with the MMSE scores in AD patients (Chu et al., 2021). Nevertheless, in our study, there was no significant correlation observed between MMSE scores and the volumes of bilateral hippocampal subfields, potentially indicating the MMSE’s reduced sensitivity in identifying MCI.

The present study has several limitations. First, the sample size was relatively small, and the study was conducted at a single center. Expanding the sample size and incorporating data from multiple centers would allow for the creation of independent validation sets. Second, the study exclusively utilized structural MRI data; a better classification accuracy of approximately 0.9 could be achieved if other imaging metrics such as abnormal functional connection mode of hippocampus subfields, default mode network (DMN) modulation, and quantitative analysis of amyloid and tau using PET molecular tracers were included and more classifiers were implemented (Zhou, 2021). Future research should consider the integration of multimodal imaging data and other domain metrics, such as multiomic biospecimens and genetic data. Third, this study was cross-sectional in nature; further longitudinal studies are needed in future to observe the dynamic changes in hippocampal atrophy patterns in AD spectrum diseases.

## Conclusion

Our findings indicate that multiple hippocampal subfield volumes undergo changes in individuals with aMCI. These alterations in hippocampal subfield volumes may serve as predictors of aMCI and appear to play a pivotal role in overall cognitive performance. Furthermore, ongoing refinement in the pattern of subfield atrophy holds promise for advancing precision medicine for MCI and AD patients, facilitating the identification of those MCI individuals at the highest risk of progressing to dementia.

## Data availability statement

The raw data supporting the conclusions of this article will be made available by the authors, without undue reservation.

## Ethics statement

The studies involving humans were approved by the Ethics Committee of Zhejiang Provincial People’s Hospital (2012KY002). The studies were conducted in accordance with the local legislation and institutional requirements. The participants provided their written informed consent to participate in this study.

## Author contributions

QF: Formal analysis, Investigation, Methodology, Writing – original draft, Writing – review & editing. LW: Methodology, Validation, Writing – original draft, Writing – review & editing. XT: Methodology, Software, Writing – original draft. XG: Methodology, Validation, Writing – original draft. HH: Software, Visualization, Writing – original draft. ZL: Conceptualization, Validation, Writing – review & editing. ZD: Conceptualization, Validation, Writing – review & editing.
